# Canine Parvovirus 2C Identified in Dog Feces from Poop Bags Collected from Outdoor Waste Bins in Arizona USA, June 2022

**DOI:** 10.1155/2023/5596886

**Published:** 2023-10-04

**Authors:** Temitope O. C. Faleye, Erin M. Driver, Devin A. Bowes, Abriana Smith, Nicole A. Kaiser, Jillian M. Wright, Ainsley R. Chapman, Rolf U. Halden, Arvind Varsani, Matthew Scotch

**Affiliations:** ^1^The Biodesign Institute Center for Environmental Health Engineering, Arizona State University, Tempe, AZ, USA; ^2^School of Sustainable Engineering and the Built Environment, Arizona State University, Tempe, AZ, USA; ^3^One Water One Health, Nonprofit Project of the Arizona State University Foundation, Tempe, AZ, USA; ^4^Biodesign Center for Fundamental and Applied Microbiomics, Center for Evolution and Medicine, School of Life Sciences, Arizona State University, Tempe, AZ, USA; ^5^College of Health Solutions, Arizona State University, Tempe, AZ, USA

## Abstract

Canine parvoviruses (CPVs) are a major cause of morbidity and mortality in dogs. However, surveillance has been largely limited to clinically manifest cases, resulting in a dearth of CPV genomic information on virus type, abundance, and diversity, limiting our understanding of its evolutionary dynamics. We tested the feasibility of using dog feces in poop bags collected from outdoor waste bins as a source for environmental surveillance of CPV. After polymerase chain reaction, long-read sequencing, and bioinformatics, we identified that CPV-2c was present in Arizona, USA, in June 2022 and documented variants with amino acid substitutions 530E and 101K in NS1 and NS2, respectively. Based on publicly available sequence data in GenBank as of January 2023, the CPV genome described here represents the only CPV genome described in the USA from the 2022 season, despite news of CPV outbreak-associated fatalities in dogs in the USA. This highlights the need for more studies that document CPV complete or near complete genomes, as well as experimental studies, to further our understanding of its evolutionary process.

## 1. Introduction

Canine parvoviruses (CPVs) are a major cause of morbidity and mortality in dogs, with clinical manifestations spanning vomiting, diarrhea, myocarditis, leukopenia, and death, among others. The cost of treatment in the USA ranges from $1,000 to $2,000, and survival rates can range from 9% to 90% depending on the age of the dog, vaccination status, and quality of treatment (if any). Hence, disease-associated euthanasia is a common endpoint for many infected and symptomatic dogs [1–3]. CPVs have been described globally, yet surveillance has been largely limited to clinical cases serious enough to necessitate a visit to the veterinarian [2, 4–6]. Hence, there is a dearth of CPV sequence data from asymptomatic and/or subclinical cases available in public databases, limiting genomic epidemiology and our understanding of its evolutionary dynamics [7].

CPVs are members of the species *Protoparvovirus carnivoran*1 (alongside feline panleukopenia virus (FPV)) in the genus *Protoparvovirus*, family *Parvoviridae* [8, 9]. They are small (∼25 nm diameter), nonenveloped viruses with icosahedral capsid symmetry and a single-stranded DNA genome of ∼5 kb. The genome has two coding frames that encode four proteins (NS1, NS2, VP1, and VP2). VP1 and VP2 form the virus capsid, with VP2 being the major capsid protein. Classification into CPV, CPV-2a, CPV-2b, and CPV-2c is based on amino acid substitutions in key antigenic sites in the VP2 protein, with N/D426E being the determinant of the CPV-2c group [2].

In the USA, it is estimated that there are over 70 million dogs [10], most of which live in human residences as pets or in shelters. Daily, dog owners walk their dogs for defecation in public spaces, subsequently pick up the feces with plastic poop bags and discard the bag in designated bins or trash cans. We posited that dog feces in poop bags discarded in the trash might provide a valuable source of samples for surveillance of CPV (and other dog viruses or pathogens). In this study, we show the detection of CPV complete genome sequences from environmental samples (dog feces in poop bags discarded in the trash) by coupling complete genome long-range polymerase chain reaction (PCR) with long-read high throughput sequencing. However, more sequences are needed domestically and globally for robust genomic epidemiology of this virus.

## 2. Methods

On June 27, 2022, we collected 73 bag-wrapped fecal samples from trash cans in eight dog parks (10 from each of six parks and six and seven from the remaining two) in the City of Tempe, Maricopa County, Arizona, USA. The samples were transported to our laboratory at Biodesign Institute, Arizona State University, USA, where sample processing was done in a Class 2 biosafety cabinet. The samples were pooled by sampling site for analysis. Specifically, for each sample site, 1 g of fecal core per sample was added to a 50 ml centrifuge tube containing resuspension solution (25 ml of PCR grade water and 15 glass beads (3 mm, Cole-Parmer, USA)). All samples from the same site were added to the same 50 ml centrifuge tube containing resuspension solution; in all, we had eight pools. All fecal pools were resuspended by vortexing (Heidolph Instruments, Germany) for 20 min at 3,000 rpm and subsequently centrifuged for 20 min at 3,000 rpm and 4°C. The supernatant was transferred into a centrifugal filter 10,000 MW cutoff, concentrated to 3 ml, and stored in 1 ml aliquots at −80°C.

Viral DNA was extracted using the Qiagen viral RNA extraction kit following the manufacturer's recommendation (QIAGEN, Germantown, MD, USA). The DNA was thereafter subjected to a nested PCR assay using primers described in Mira et al. [6], with slight modifications. Specifically, instead of amplifying the genome in two overlapping fragments as described [6], we amplified the near-complete genome (∼4,600 bp; nucleotides 100–4692 relative to MF416372.1) with the outer primers (NS-Fext (5′-GACCGTTACTGACATTCGCTTC-3′) and 4835R (5′-ACCAACCACCCACACCATAACAAC-3′)) and used the first round amplicon (as template) alongside the internal primers (2161F (5′-TTGGCGTTACTCACAAAGACGTGC-3′) and NS-Rext (5′-GAAGGGTTAGTTGGTTCTCC-3′)) for a confirmatory nested-PCR assay (∼340 bp; nucleotide 2050–2390 relative to MF416372.1, which spans the end of NS1 and the beginning of VP1). Phusion plus green (ThermoFisher Scientific, Waltham, MA, USA) and GoTaq green (Promega, Madison, WI, USA) PCR master mixes were used for the first and second-round assays, respectively. Thermal cycling conditions include 94°C for 3 min, 40 cycles of 94°C for 30 s, 55°C for 30 s, and 68°C for 6 min, and finally 68°C for 10 min for the first-round assay and 94°C for 3 min, 35 cycles of 94°C for 30 s, 55°C for 30 s, and 60°C for 30 s, and finally 72°C for 10 min for the second-round assay. The second-round amplicon was Sanger sequenced using both the forward and reverse primers. The sequence data generated was used as a query in a BLASTn search of the GenBank database [11] to confirm the amplicon was from a CPV genome. For any positive pool, individual samples in the pool were resuspended and reanalyzed independently as described above with slight modification. Specifically, resuspension was done with a resuspension solution containing 5 ml of PCR-grade water.

First-round amplicons of samples positive for the second-round assay were cleaned and used for MinION library preparation (ligation sequencing kit, SQK-LSK110) according to the manufacturer's instructions. The library was sequenced on a Flongle flow cell for 1 hr, and base calling was done using Guppy in MinKNOW. The FASTQ reads were merged using merge-read-libraries v1.0.1, trimmed using Porechop v0.2.4, and assembled by template-guided-assembly using Minimap2 v2.24. Variant analysis was done using the “find variation/SNPs” plugin in Geneious prime v2022.2.2 [12]. The homopolymer regions in the consensus genome were manually scanned and polished using the bam file generated by Minimap2 alongside an alignment of the genome to the top three hits in GenBank.

The consensus genome was used as a query in a BLASTn search of the GenBank database [11], and the top 250 hits were downloaded to make a local database to which the genome recovered in this study was added. The database was multiple sequence aligned (MSA) using the MAFFT online server [13]. Phylogenetic analysis was done using MEGA X [14], and all phylogenetic trees were visualized and annotated using iTOL v6 [15]. Neighbor-Joining trees (1,000 bootstrap replicates) of complete contig, NS1 and VP2 were made, and sequences that clustered with that described in this study with >65% bootstrap support were assembled into a second database alongside that described in this study. The second database was subjected to MSA, and maximum-likelihood (ML) phylogenetic trees (GTR model and 1,000 bootstrap replicates) of the complete contig, NS1 and VP2 were done in MEGA X [14]. The alignment file was also subjected to amino acid conservation analysis in BioEdit v7.0.5.3 [16]. Please see *Supplementary 1* for a schematic representation of the workflow for this study.

## 3. Results

The ∼340 bp amplicon product of the second-round assay was detected in one of the eight pools and only in one of the seven samples in the pool. A BLASTn search using the Sanger sequencing data (accession number OQ266794) as query showed the ∼340 bp contig was 100% identical (with 100% query cover) to the top 13 hits in GenBank, which were annotated as CPVs except MN451692 which was annotated as an FPV and was the topmost hit.

Precisely, 16,124 raw reads were generated from the Flongle flow cell. Post trimming, 16,166 raw reads remained, 11,473 of which were mapped to MN451692.1 to generate the consensus genome (accession number OQ266793). Variant analysis showed amino acid substitutions in NS1 (E530K, F544Y, K572E), NS2 (E101K) and VP2 (R46K, D300G, H305Y, N426E, and T440A) relative to MN451692 (*Supplementary 2*). All variant sites with the amino acid substitutions listed above had a depth of coverage >11,000x and frequency >80% except for NS1 E530K, which though had 11,102x coverage, had a variant frequency of 56.90% (*Supplementary 2*). This suggests contigs with both glutamic acid (E) and lysine (K) at NS1-530 were both present in the pool.

**Figure 1:**
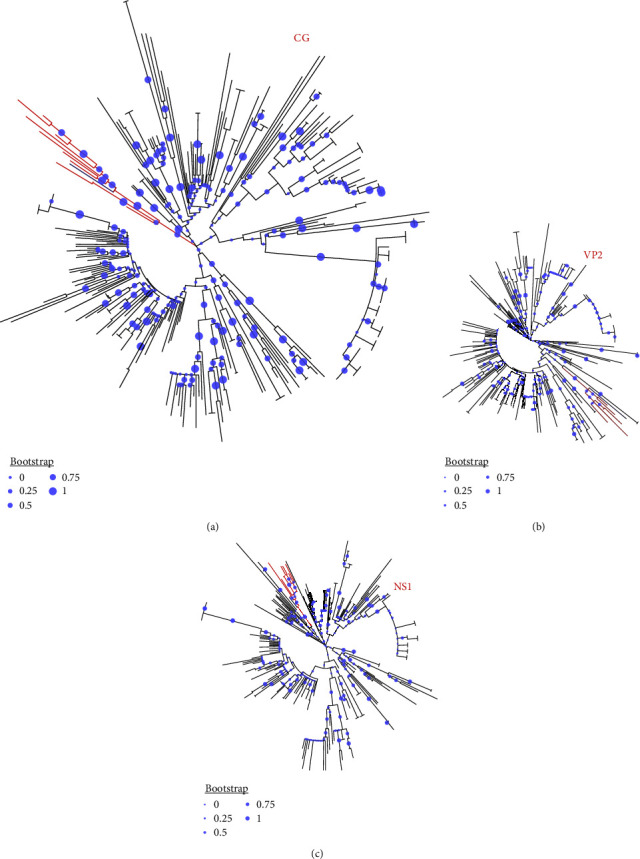
Phylogenetic trees of the top 250 GenBank hits of DP8. (a), (b), and (c) Show trees for the complete genome, VP2, and NS1, respectively. DP8 is highlighted in blue, while the 10 sequences that cluster with it are highlighted in red.

**Figure 2:**
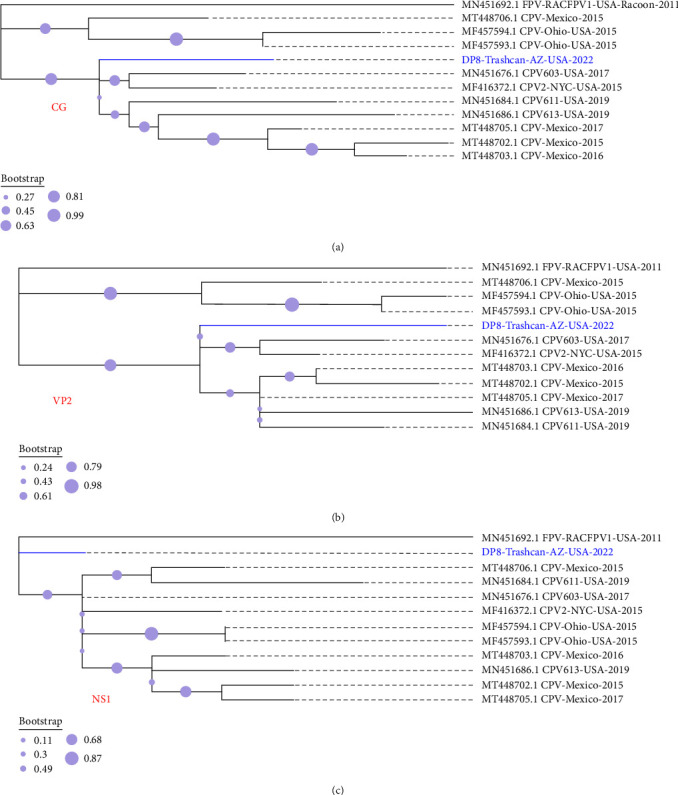
Maximum-likelihood trees of the 10 sequences that cluster with DP8 in Figure 1(a), 1(b), and 1(c) alongside FPV (highlighted in bold). (a), (b), and (c) Show trees for complete genome, VP2, and NS1, respectively.

**Figure 3:**
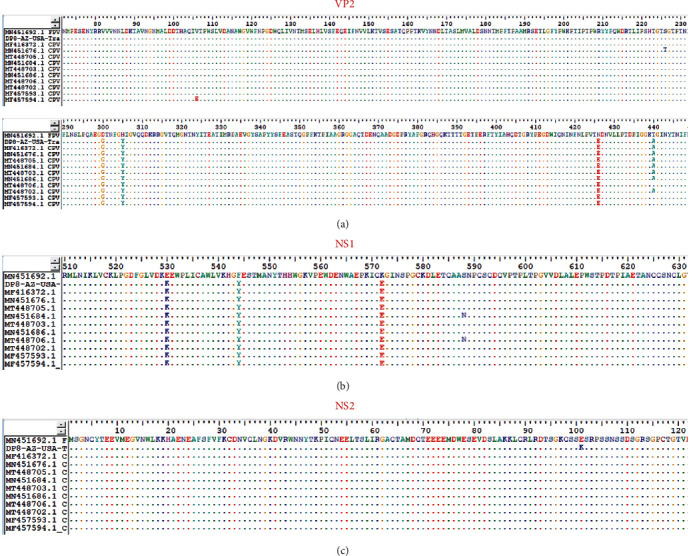
Conservation analysis of DP8 amino acid sequence alongside sequences analyzed in Figures 2(a), 2(b), and 2(c). (a), (b), and (c) Show alignments for VP2, NS1, and NS2, respectively, with MN451692 as the reference.

The consensus genome (4269nt, GC% = 36%, subsequently referred to as DP8) encodes all four ORFs (NS1, NS2, VP1, and VP2). A BLASTn search of the GenBank database showed all top 250 hits (100% query cover) were >99% identical to DP8. Phylogenetic analysis of complete contig, NS1 and VP2 revealed 10 sequences that clustered (>65% bootstrap support) with DP8 in either one or more of the trees (Figure 1(a)–1(c)). ML trees of these 10 sequences (all from the USA and Mexico) alongside DP8 and MN451692 (FPV) confirmed DP8 clusters with them (Figure 2(a)–2(c)). Amino acid conservation analysis of the VP2 protein (Figure 3(a)) showed that all 10 sequences alongside DP8 had the N/D426E substitution, typing them all as CPV-2c. Furthermore, DP8 had E530K substitution in the NS1 protein (Figure 3(b)), and variant analysis showed that 530K was only present in 56.90% of mapped reads from the DP8 pool, suggesting that 530E was also present (*Supplementary 2*). DP8, however, differed from all 10 in that it also had E101K substitution in the NS2 protein (Figure 3(c)).

## 4. Discussion/Conclusions

We tested the feasibility of sampling dog feces in plastic poop collection bags discarded in designated outdoor waste bins as a valuable source for surveillance of CPV (and other dog viruses or pathogens), and our findings confirm its suitability. This approach provides a noninvasive alternative for CPV genomic surveillance that also provides an avenue for exploring virus diversity year-round, especially in cases where infection might not have produced clinical manifestations or symptoms were mild enough not to necessitate visits to the veterinarian. Furthermore, genomic data from this source might provide the insight necessary to better understand the evolution of CPV and consequent improvement in the sensitivity of diagnostics, thereby reducing false negative detections that sometimes plague CPV diagnosis [17].

Based on publicly available sequence data in GenBank as of January, 2023, the CPV genome described here (DP8) represents the only CPV genome described in the USA from the 2022 season, despite news of CPV outbreak-associated fatalities in dogs in the USA [18]. In this study, we show CPV-2c was present in Arizona, USA, in June 2022. It is important to note that the VP2 amino acid substitutions A5G and Q370R associated with CPV-2c variants circulating in Asia were absent in DP8 (*Supplementary 3*). Furthermore, DP8 clustered with variants previously detected in the USA and Mexico (Figures 1 and 2). It is, therefore, likely that DP8 belongs to a lineage circulating in North America. We also show the presence of variants with amino acid substitutions (530E and 101K in NS1 and NS2, respectively) in the population (Figure 3 and *Supplementary 2*). Since no genomic data are currently publicly available from the 2022 Michigan, USA CPV-2c outbreak [18], it is not clear how similar the genome described here is to those associated with the dozens of CPV-associated dog fatalities in Michigan, USA. Furthermore, most [2, 4–6] CPV genomic epidemiology studies focus on VP2 sequencing due to its value for virus typing. Hence, there is limited information on the evolutionary dynamics of the NS1 and NS2 genes. Consequently, it is not clear how the substitution we have found in NS1 and NS2 might impact the phenotype of DP8. This highlights the need for more studies that document CPV complete or near complete genomes (at least complete coding sequence of NS1 and VP) as well as experimental studies to further our understanding of the evolutionary dynamics of the NS1 and NS2 proteins.

In this study, we coupled complete genome (single-contig) long-range PCR with long-read high throughput sequencing and showed the utility of this workflow for environmental surveillance using dog feces in plastic poop collection bags discarded in outdoor waste bins. We are aware that the workflow described in this study should support genomic epidemiology of CPV (and other viruses) in other sample types, and our preliminary analysis (unpublished) applying it to municipal wastewater confirms that wastewater-based genomic epidemiology of CPV is feasible and might further contribute to our understanding of CPV dynamics on a population scale.

## Data Availability

The data presented in this study are openly available in SRA under accession numbers PRJNA923767 and in GenBank under accession number OQ266793 and OQ266794.

## Supplementary Materials

Supplementary 1 Schematic representation of the workflow for this study.

Supplementary 2 Variant profile of canine parvovirus DP8 described in this study.

Supplementary 3 Conservation analysis of DP8 complete VP2 amino acid sequence alongside sequences analyzed in Figure 2(a).

## References

[1] Kelman M., Ward M. P., Barrs V. R., Norris J. M. (2019). The geographic distribution and financial impact of canine parvovirus in Australia. *Transboundary and Emerging Diseases*.

[2] Miranda C., Thompson G. (2016). Canine parvovirus: the worldwide occurrence of antigenic variants. *Journal of General Virology*.

[3] Horecka K., Porter S., Amirian E. S., Jefferson E. (2020). A decade of treatment of canine parvovirus in an animal shelter: a retrospective study. *Animals*.

[4] Zhou P., Zeng W., Zhang X., Li S. (2017). The genetic evolution of canine parvovirus—a new perspective. *PLOS ONE*.

[5] Hoelzer K., Shackelton L. A., Parrish C. R., Holmes E. C. (2008). Phylogenetic analysis reveals the emergence, evolution and dispersal of carnivore parvoviruses. *Journal of General Virology*.

[6] Mira F., Purpari G., Lorusso E. (2018). Introduction of Asian canine parvovirus in Europe through dog importation. *Transboundary and Emerging Diseases*.

[7] Voorhees I. E. H., Lee H., Allison A. B. (2019). Limited intrahost diversity and background evolution accompany 40 years of canine parvovirus host adaptation and spread. *Journal of Virology*.

[8] Pénzes J. J., Söderlund-Venermo M., Canuti M. (2020). Reorganizing the family *Parvoviridae*: a revised taxonomy independent of the canonical approach based on host association. *Archives of Virology*.

[9] Cotmore S. F., Agbandje-McKenna M., Chiorini J. A. (2014). The family *Parvoviridae*. *Archives of Virology*.

[10] Wasik B. R., Voorhees I. E. H., Parrish C. R. (2021). Canine and feline influenza. *Cold Spring Harbor Perspectives in Medicine*.

[11] Sayers E. W., Cavanaugh M., Clark K. (2021). GenBank. *Nucleic Acids Research*.

[12] Kearse M., Moir R., Wilson A. (2012). Geneious basic: an integrated and extendable desktop software platform for the organization and analysis of sequence data. *Bioinformatics*.

[13] Katoh K., Rozewicki J., Yamada K. D. (2019). MAFFT online service: multiple sequence alignment, interactive sequence choice and visualization. *Briefings in Bioinformatics*.

[14] Kumar S., Stecher G., Li M., Knyaz C., Tamura K. (2018). MEGA X: molecular evolutionary genetics analysis across computing platforms. *Molecular Biology and Evolution*.

[15] Letunic I., Bork P. (2021). Interactive tree of life (iTOL) v5: an online tool for phylogenetic tree display and annotation. *Nucleic Acids Research*.

[16] Hall T. A. (1999). BioEdit: a user-friendly biological sequence alignment editor and analysis program for windows 95/98/NT. *Nucleic Acids Symposium Series*.

[17] Walter-Weingärtner J., Bergmann M., Weber K., Truyen U., Muresan C., Hartmann K. (2021). Comparison of eight commercially available faecal point-of-care tests for detection of canine parvovirus antigen. *Viruses*.

[18] MSU-VDL (2022). Parvo-like illness reported in northern michigan dogs: updates. https://cvm.msu.edu/vdl/news/2022/parvo-like-illness-reported-in-northern-michigan-dogs-updates.

