# Supplementary material for: Canine Parvovirus 2C Identified in Dog Feces from Poop Bags Collected from Outdoor Waste Bins in Arizona USA, June 2022
Source: Transbound Emerg Dis. Author manuscript; Available in PMC 2024 Jul 9. (PMC11232495; doi:10.1155/2023/5596886)
Supplement: Supplementary 2 — Variant profile of canine parvovirus DP8 described in this study. [file NIHMS1990086-supplement-Supplementary_2.docx]

Table S1: Variant profile of Canine parvovirus DP8 described in this study.

| Name | Type | Minimum | Maximum | Length | # Intervals | Amino Acid Change | CDS Position | Change | Codon Change | Coverage | Polymorphism Type | Protein Effect | Variant Frequency | Variant P-Value (approximate) |
| --- | --- | --- | --- | --- | --- | --- | --- | --- | --- | --- | --- | --- | --- | --- |
| T | Polymorphism | 4266 | 4266 | 1 | 1 |  | 2181 | C -> T | TAC -> TAT | 10026 | SNP (transition) | None | 98.90% | 0 |
| T | Polymorphism | 4266 | 4266 | 1 | 1 |  | 1752 | C -> T | TAC -> TAT | 10026 | SNP (transition) | None | 98.90% | 0 |
| G | Polymorphism | 4230 | 4230 | 1 | 1 |  | 2145 | A -> G | GTA -> GTG | 11140 | SNP (transition) | None | 84.70% | 0 |
| G | Polymorphism | 4230 | 4230 | 1 | 1 |  | 1716 | A -> G | GTA -> GTG | 11140 | SNP (transition) | None | 84.70% | 0 |
| A | Polymorphism | 4014 | 4014 | 1 | 1 |  | 1929 | G -> A | GTG -> GTA | 11286 | SNP (transition) | None | 76.40% | 0 |
| A | Polymorphism | 4014 | 4014 | 1 | 1 |  | 1500 | G -> A | GTG -> GTA | 11286 | SNP (transition) | None | 76.40% | 0 |
| A | Polymorphism | 3882 | 3882 | 1 | 1 |  | 1797 | C -> A | GCC -> GCA | 11289 | SNP (transversion) | None | 94.20% | 0 |
| A | Polymorphism | 3882 | 3882 | 1 | 1 |  | 1368 | C -> A | GCC -> GCA | 11289 | SNP (transversion) | None | 94.20% | 0 |
| G | Polymorphism | 3832 | 3832 | 1 | 1 | T -> A | 1747 | A -> G | ACA -> GCA | 11274 | SNP (transition) | Substitution | 86.50% | 0 |
| G | Polymorphism | 3832 | 3832 | 1 | 1 | T -> A | 1318 | A -> G | ACA -> GCA | 11274 | SNP (transition) | Substitution | 86.50% | 0 |
| A | Polymorphism | 3804 | 3804 | 1 | 1 |  | 1719 | G -> A | TTG -> TTA | 11265 | SNP (transition) | None | 90.90% | 0 |
| A | Polymorphism | 3804 | 3804 | 1 | 1 |  | 1290 | G -> A | TTG -> TTA | 11265 | SNP (transition) | None | 90.90% | 0 |
| GAA | Polymorphism | 3790 | 3792 | 2 | 2 | N -> E | 1705 | AAT -> GAA | AAT -> GAA | 11264 | Substitution | Substitution | 89.30% | 0 |
| GAA | Polymorphism | 3790 | 3792 | 2 | 2 | N -> E | 1276 | AAT -> GAA | AAT -> GAA | 11264 | Substitution | Substitution | 89.30% | 0 |
|  | Polymorphism | 3671 | 3671 | 1 | 1 |  | 1586 | (A)6 -> (A)5 | | 11233 | Deletion (tandem repeat) | Frame Shift | 33.40% | 2.10E-245 |
|  | Polymorphism | 3671 | 3671 | 1 | 1 |  | 1157 | (A)6 -> (A)5 | | 11233 | Deletion (tandem repeat) | Frame Shift | 33.40% | 2.10E-245 |
|  | Polymorphism | 3596 | 3596 | 1 | 1 |  | 1511 | (G)7 -> (G)6 | | 11207 | Deletion (tandem repeat) | Frame Shift | 74.10% | 0 |
|  | Polymorphism | 3596 | 3596 | 1 | 1 |  | 1082 | (G)7 -> (G)6 | | 11207 | Deletion (tandem repeat) | Frame Shift | 74.10% | 0 |
| C | Polymorphism | 3540 | 3540 | 1 | 1 |  | 1455 | T -> C | TAT -> TAC | 11193 | SNP (transition) | None | 91.70% | 0 |
| C | Polymorphism | 3540 | 3540 | 1 | 1 |  | 1026 | T -> C | TAT -> TAC | 11193 | SNP (transition) | None | 91.70% | 0 |
| T | Polymorphism | 3427 | 3427 | 1 | 1 | H -> Y | 1342 | C -> T | CAT -> TAT | 11188 | SNP (transition) | Substitution | 87.30% | 0 |
| T | Polymorphism | 3427 | 3427 | 1 | 1 | H -> Y | 913 | C -> T | CAT -> TAT | 11188 | SNP (transition) | Substitution | 87.30% | 0 |
| G | Polymorphism | 3413 | 3413 | 1 | 1 | D -> G | 1328 | A -> G | GAT -> GGT | 11193 | SNP (transition) | Substitution | 83.80% | 0 |
| G | Polymorphism | 3413 | 3413 | 1 | 1 | D -> G | 899 | A -> G | GAT -> GGT | 11193 | SNP (transition) | Substitution | 83.80% | 0 |
|  | Polymorphism | 3310 | 3311 | 2 | 1 |  | 1225 | (TT)4 -> (TT)3 | | 11186 | Deletion (tandem repeat) | Frame Shift | 45.80% | 0 |
|  | Polymorphism | 3310 | 3311 | 2 | 1 |  | 796 | (TT)4 -> (TT)3 | | 11186 | Deletion (tandem repeat) | Frame Shift | 45.80% | 0 |
| G | Polymorphism | 3117 | 3117 | 1 | 1 |  | 1032 | A -> G | AAA -> AAG | 11156 | SNP (transition) | None | 85.60% | 0 |
| G | Polymorphism | 3117 | 3117 | 1 | 1 |  | 603 | A -> G | AAA -> AAG | 11156 | SNP (transition) | None | 85.60% | 0 |
| T | Polymorphism | 3024 | 3024 | 1 | 1 |  | 939 | C -> T | ACC -> ACT | 11156 | SNP (transition) | None | 93.40% | 0 |
| T | Polymorphism | 3024 | 3024 | 1 | 1 |  | 510 | C -> T | ACC -> ACT | 11156 | SNP (transition) | None | 93.40% | 0 |
| G | Polymorphism | 2964 | 2964 | 1 | 1 |  | 879 | A -> G | TTA -> TTG | 11149 | SNP (transition) | None | 90.60% | 0 |
| G | Polymorphism | 2964 | 2964 | 1 | 1 |  | 450 | A -> G | TTA -> TTG | 11149 | SNP (transition) | None | 90.60% | 0 |
| A | Polymorphism | 2658 | 2658 | 1 | 1 |  | 573 | G -> A | CAG -> CAA | 11056 | SNP (transition) | None | 94.80% | 0 |
| A | Polymorphism | 2658 | 2658 | 1 | 1 |  | 144 | G -> A | CAG -> CAA | 11056 | SNP (transition) | None | 94.80% | 0 |
| A | Polymorphism | 2658 | 2658 | 1 | 1 | R -> K | 137 | G -> A | AGA -> AAA | 11056 | SNP (transition) | Substitution | 94.80% | 0 |
| nonstructural protein SAT CDS | CDS | 2522 | 2728 | 207 | 1 |  |  |  |  |  |  |  |  |  |
| VP2 CDS | CDS | 2515 | 4269 | 1755 | 1 |  |  |  |  |  |  |  |  |  |
|  | Polymorphism | 2493 | 2493 | 1 | 1 |  | 408 | (A)5 -> (A)4 | | 11051 | Deletion (tandem repeat) | Frame Shift | 28.90% | 1.30E-111 |
| A | Polymorphism | 2487 | 2487 | 1 | 1 |  | 402 | G -> A | GGG -> GGA | 11050 | SNP (transition) | None | 94.20% | 0 |
|  | Polymorphism | 2464 | 2464 | 1 | 1 |  | 379 | (A)13 -> (A)12 | | 11049 | Deletion (tandem repeat) | Frame Shift | 65.30% | 0 |
|  | Polymorphism | 2463 | 2463 | 1 | 1 |  | 378 | (A)13 -> (A)12 | | 11049 | Deletion (tandem repeat) | Frame Shift | 74.20% | 0 |
|  | Polymorphism | 2350 | 2350 | 1 | 1 |  | 265 | (A)5 -> (A)4 | | 11038 | Deletion (tandem repeat) | Frame Shift | 30.20% | 3.90E-33 |
|  | Polymorphism | 2338 | 2338 | 1 | 1 |  | 253 | (T)7 -> (T)6 | | 11032 | Deletion (tandem repeat) | Frame Shift | 30.00% | 1.90E-303 |
|  | Polymorphism | 2337 | 2337 | 1 | 1 |  | 252 | (T)7 -> (T)6 | | 11031 | Deletion (tandem repeat) | Frame Shift | 51.10% | 0 |
|  | Polymorphism | 2317 | 2317 | 1 | 1 |  | 232 | (G)8 -> (G)7 | | 11029 | Deletion (tandem repeat) | Frame Shift | 27.50% | 1.20E-80 |
|  | Polymorphism | 2315 | 2316 | 2 | 1 |  | 230 | (GG)4 -> (GG)3 | | 11028 -> 11030 | Deletion (tandem repeat) | Frame Shift | 54.60% | 0 |
|  | Polymorphism | 2106 | 2106 | 1 | 1 |  |  | (T)7 -> (T)6 | | 11006 | Deletion (tandem repeat) | | 35.10% | 0 |
|  | Polymorphism | 2066 | 2066 | 1 | 1 |  |  | (G)6 -> (G)5 | | 11008 | Deletion (tandem repeat) | | 51.90% | 0 |
| VP1 CDS | CDS | 2014 | 4269 | 2184 | 2 |  |  |  |  |  |  |  |  |  |
| A | Polymorphism | 1773 | 1773 | 1 | 1 |  | 1773 | G -> A | CAG -> CAA | 11056 | SNP (transition) | None | 89.00% | 0 |
| A | Polymorphism | 1773 | 1773 | 1 | 1 | E -> K | 301 | G -> A | GAG -> AAG | 11056 | SNP (transition) | Substitution | 89.00% | 0 |
| G | Polymorphism | 1714 | 1714 | 1 | 1 | K -> E | 1714 | A -> G | AAA -> GAA | 11070 | SNP (transition) | Substitution | 87.90% | 0 |
| A | Polymorphism | 1631 | 1631 | 1 | 1 | F -> Y | 1631 | T -> A | TTT -> TAT | 11088 | SNP (transversion) | Substitution | 90.70% | 0 |
| A | Polymorphism | 1588 | 1588 | 1 | 1 | E -> K | 1588 | G -> A | GAA -> AAA | 11102 | SNP (transition) | Substitution | 56.90% | 0 |
|  | Polymorphism | 1588 | 1588 | 1 | 1 |  | 1588 | #NAME? |  | 11102 | Deletion | Frame Shift | 40.50% | 0 |
| A | Polymorphism | 1551 | 1551 | 1 | 1 |  | 1551 | G -> A | GTG -> GTA | 11114 | SNP (transition) | None | 90.20% | 0 |
| A | Polymorphism | 1455 | 1455 | 1 | 1 |  | 1455 | G -> A | ACG -> ACA | 11133 | SNP (transition) | None | 95.60% | 0 |
|  | Polymorphism | 1100 | 1100 | 1 | 1 |  | 1100 | (T)5 -> (T)4 | | 11261 | Deletion (tandem repeat) | Frame Shift | 28.30% | 2.40E-100 |
|  | Polymorphism | 972 | 972 | 1 | 1 |  | 972 | (A)6 -> (A)5 | | 11285 | Deletion (tandem repeat) | Frame Shift | 34.50% | 1.80E-285 |
|  | Polymorphism | 838 | 838 | 1 | 1 |  | 838 | (A)5 -> (A)4 | | 11310 | Deletion (tandem repeat) | Frame Shift | 27.80% | 2.10E-11 |
| C | Polymorphism | 693 | 693 | 1 | 1 |  | 693 | T -> C | AGT -> AGC | 11397 | SNP (transition) | None | 91.30% | 0 |
|  | Polymorphism | 684 | 684 | 1 | 1 |  | 684 | (T)6 -> (T)5 | | 11449 | Deletion (tandem repeat) | Frame Shift | 28.60% | 8.30E-260 |
|  | Polymorphism | 649 | 649 | 1 | 1 |  | 649 | (A)7 -> (A)6 | | 11473 | Deletion (tandem repeat) | Frame Shift | 45.60% | 0 |
| T | Polymorphism | 640 | 640 | 1 | 1 |  | 640 | C -> T | CTA -> TTA | 11456 | SNP (transition) | None | 70.00% | 0 |
|  | Polymorphism | 640 | 640 | 1 | 1 |  | 640 | -C |  | 11456 | Deletion | Frame Shift | 26.60% | 1.70E-188 |
|  | Polymorphism | 585 | 585 | 1 | 1 |  | 585 | (A)7 -> (A)6 | | 9333 | Deletion (tandem repeat) | Frame Shift | 38.30% | 0 |
|  | Polymorphism | 299 | 299 | 1 | 1 |  | 299 | (T)5 -> (T)4 | | 9306 | Deletion (tandem repeat) | Frame Shift | 30.40% | 1.30E-125 |
|  | Polymorphism | 256 | 256 | 1 | 1 |  | 256 | (A)5 -> (A)4 | | 9288 | Deletion (tandem repeat) | Frame Shift | 27.50% | 4.50E-69 |
|  | Polymorphism | 256 | 256 | 1 | 1 |  | 256 | (A)5 -> (A)4 | | 9288 | Deletion (tandem repeat) | Frame Shift | 27.50% | 4.50E-69 |
|  | Polymorphism | 36 | 36 | 1 | 1 |  | 36 | (G)3 -> (G)2 | | 9091 | Deletion (tandem repeat) | Frame Shift | 37.80% | 2.20E-157 |
|  | Polymorphism | 36 | 36 | 1 | 1 |  | 36 | (G)3 -> (G)2 | | 9091 | Deletion (tandem repeat) | Frame Shift | 37.80% | 2.20E-157 |
| nonstructural protein 1 CDS | CDS | 1 | 2007 | 2007 | 1 |  |  |  |  |  |  |  |  |  |
| nonstructural protein 2 CDS | CDS | 1 | 1970 | 498 | 2 |  |  |  |  |  |  |  |  |  |
