# Supplementary figures and images for: Canine Parvovirus 2C Identified in Dog Feces from Poop Bags Collected from Outdoor Waste Bins in Arizona USA, June 2022
Source: Transbound Emerg Dis. Author manuscript; Available in PMC 2024 Jul 9. (PMC11232495; doi:10.1155/2023/5596886)

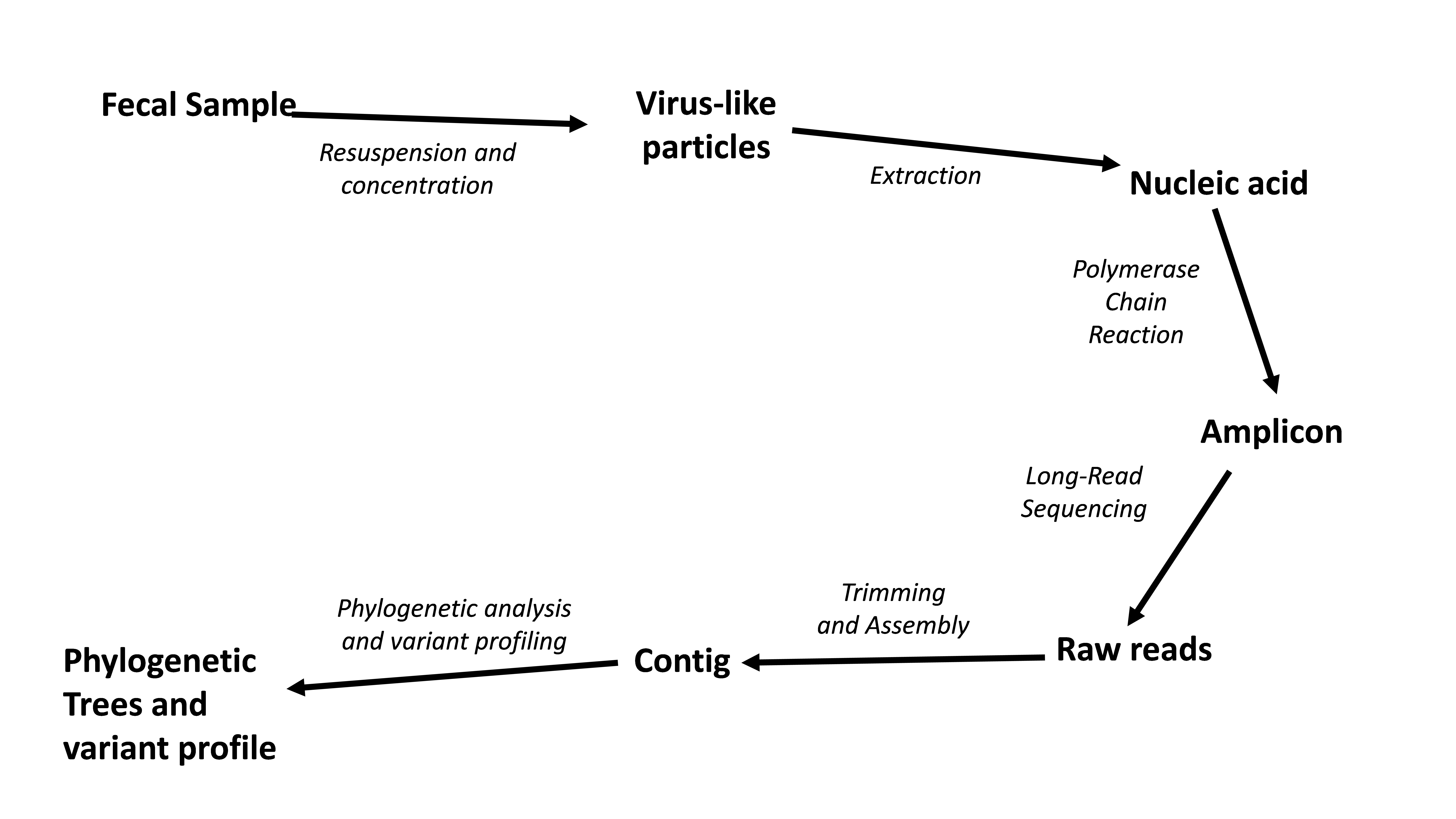

Supplement: Supplementary 1 — Schematic representation of the workflow for this study. [file NIHMS1990086-supplement-Supplementary_1.jpg]

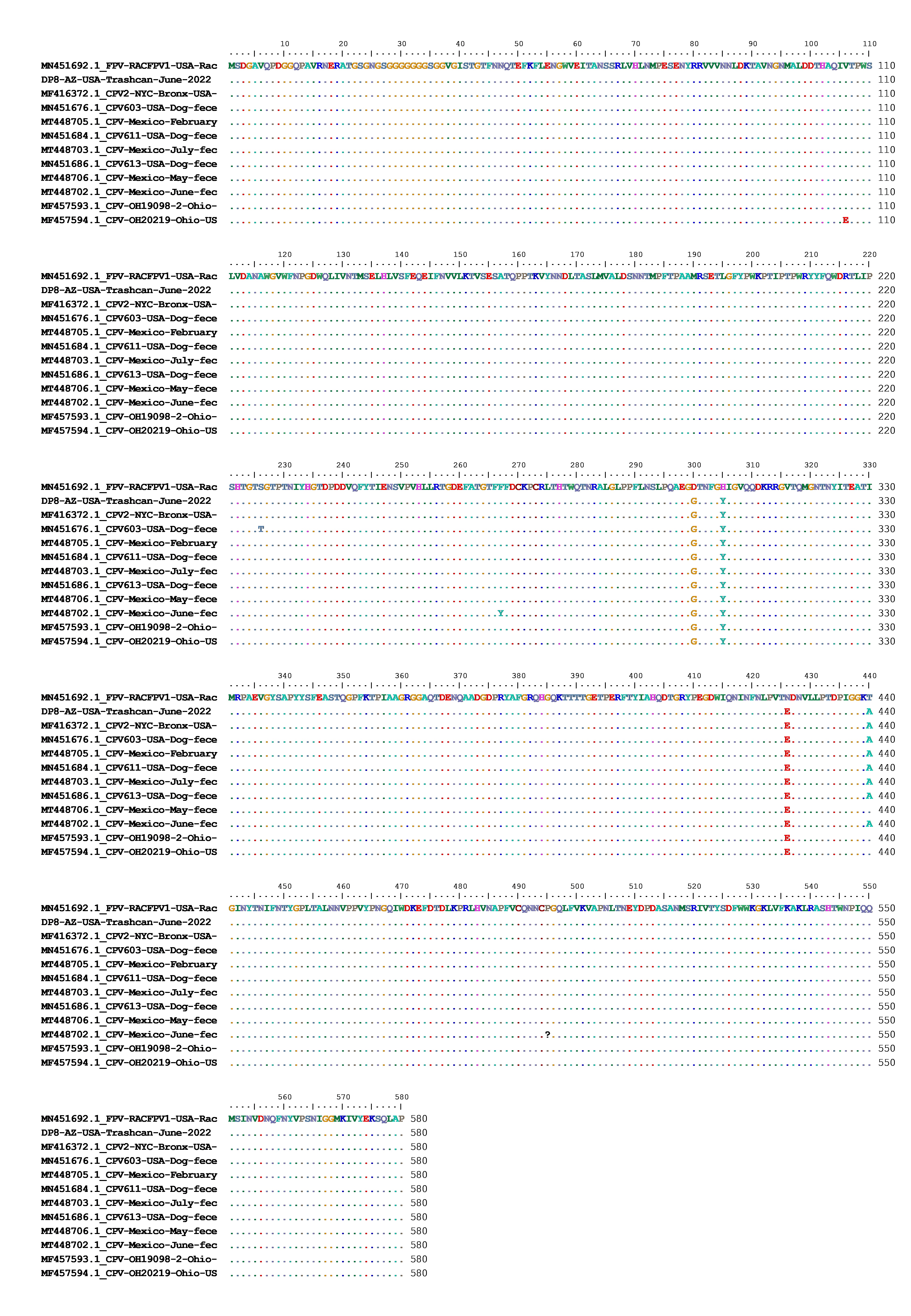

Supplement: Supplementary 3 — Conservation analysis of DP8 complete VP2 amino acid sequence alongside sequences analyzed in Figure 2(a). [file NIHMS1990086-supplement-Supplementary_3.jpg]
